# Food Environment Surrounding Public Transportation Stops and Stations in a Brazilian Metropolis

**DOI:** 10.1007/s11524-026-01142-7

**Published:** 2026-09-08

**Authors:** Luana Lara Rocha, Letícia Lopes Vieira, Gilmar Junio Moreira Tomaz, Millena Campos, Larissa Loures Mendes

**Affiliations:** 1 Study, Research, and Practice Group on Food Environment and Health (GEPPAAS), Department of Nutrition, School of Nursing, Federal University of Minas Gerais (UFMG), Belo Horizonte, Brazil; 2 Department of Preventive and Social Medicine, Faculty of Medicine, Federal University of Minas Gerais (UFMG), Belo Horizonte, Minas Gerais Brazil

**Keywords:** Food environment, Food access, Public transportation, Urban health

## Abstract

Public transportation facilities are high-traffic urban spaces that may shape food access and dietary choices, yet their surrounding food environments remain understudied, particularly in socially vulnerable settings. This study characterized the food environment surrounding public transportation facilities in Belo Horizonte, Brazil. We conducted an ecological study using georeferenced data from 47 bus stations, 47,003 bus stops, and 18 subway stations in 2019. Food retail establishments and public food and nutrition security facilities (PFNSFs) were mapped within 500-m buffers and analyzed according to the health vulnerability index. Ultraprocessed food retailers predominated across all transportation facilities, whereas outlets selling unprocessed or minimally processed foods were less common. PFNSFs were virtually absent around bus stations and bus stops, including in highly vulnerable areas. These findings highlight public transportation facilities as strategic settings for urban food policies aimed at expanding access to healthy foods and reducing food environment inequalities.

## Introduction

The food environment is a key determinant of dietary behaviors and health, shaped by the physical, economic, political, and sociocultural conditions that influence food access and choices [1, 2]. Ensuring access to healthy foods is central to food and nutrition security and to the promotion of health equity, particularly in urban settings where the distribution of food retailers is uneven [3, 4]. Public transportation facilities represent strategic urban spaces because they connect different neighborhoods and are used daily by large numbers of people, especially those living in socially vulnerable areas who rely heavily on public transit [5]. Consequently, the food environment surrounding these facilities may influence food purchasing and consumption during daily commuting [2].

Evidence from Brazilian cities suggests that transportation facilities are predominantly surrounded by establishments selling ultraprocessed foods, while healthier options are less available and often less affordable [2, 6]. Similarly, studies in Belo Horizonte have reported a predominance of ultraprocessed food retailers in high pedestrian traffic areas and socially vulnerable neighborhoods, a pattern associated with unhealthy diets and increased risk of noncommunicable diseases [7–13]. However, little is known about how food environments surrounding public transportation facilities vary according to social vulnerability, particularly in large urban centers.

This study aimed to characterize the food environment surrounding public transportation facilities in Belo Horizonte, Brazil, according to the health vulnerability index. We hypothesized that facilities located in more socially vulnerable areas would have lower availability of healthy food outlets and greater availability of establishments selling ultraprocessed foods. By integrating urban mobility and social vulnerability into the assessment of the food environment, this study provides evidence to inform urban health and food policy strategies.

## Methods

This ecological study was conducted in Belo Horizonte, Brazil, in 2019. The city has approximately 2.4 million inhabitants and an extensive public transportation network comprising conventional buses, bus rapid transit (BRT), and subway services [14, 15].

The units of analysis were the areas surrounding all public transportation facilities, located within the city’s 5017 census tracts, including 47 bus stations, 47,003 bus stops, and 18 subway stations obtained from the city’s metadata catalog (https://geonetwork.pbh.gov.br/geonetwork/srv/por/catalog.search#/home). Food retail establishments (*n* = 23,384) were identified from the Minas Gerais State Department of Finance database (2019), georeferenced according to address, and classified following the Brazilian Interministerial Chamber of Food and Nutrition Security (CAISAN) into three groups: establishments predominantly selling unprocessed or minimally processed foods (fish markets, fruit and vegetable shops, and butcher shops), mixed food retailers (hypermarkets, supermarkets, minimarkets and groceries, restaurants, bakeries, and dairy and cold cuts stores), and establishments predominantly selling ultraprocessed foods (snack bars, bars, and candy stores or confectioneries) [16, 17]. Public food and nutrition security facilities (PFNSFs) (*n* = 124), including municipal food markets, farmers’ markets, and other public food access initiatives, were identified from the Belo Horizonte City Hall database [18].

Social vulnerability was assessed using the health vulnerability index (HVI), an official indicator developed by the Belo Horizonte Municipal Government based on the 2010 Brazilian Demographic Census [19]. The HVI comprises eight sanitation and socioeconomic indicators aggregated at the census tract level, with higher values indicating greater health vulnerability. Census tracts were classified as low, medium, high, or very high vulnerability; high and very high categories were combined for analysis. HVI information was available for 3830 census tracts.

A 500-m circular buffer was created around each transportation facility to identify nearby food retail establishments and PFNSF, following previous food environment studies [20–22]. Descriptive analyses included frequencies, means, and 95% confidence intervals (95% CIs). Differences between groups were assessed by examining confidence interval overlap, with nonoverlapping intervals considered indicative of statistically significant differences. Spatial analyses were conducted using QGIS 3.22 and statistical analyses using Stata 14.0.

## Results

A total of 23,508 food retail establishments were identified within the 500-m buffers surrounding 47,068 public transportation facilities in Belo Horizonte. According to the HVI, 34 bus stations and 16 subway stations were located in low-vulnerability areas, whereas 13 bus stations were located in high or very high vulnerability areas. Among bus stops, 1312 were located in low-, 1432 in medium-, and 1009 in high or very high-vulnerability areas.

Establishments predominantly selling ultraprocessed foods represented the most common food retail category surrounding all public transportation facilities, whereas establishments selling unprocessed or minimally processed foods and PFNSF were consistently less frequent (Table 1). Around bus stations, mean densities of ultraprocessed food retailers reached 23.19 (95% CI 14.08–32.31), compared with 14.94 (95% CI 9.69–20.19) for mixed food retailers and 2.87 (95% CI 1.32–4.42) for establishments selling unprocessed or minimally processed foods. This pattern was also observed around bus stops (14.21, 95% CI 13.67–14.75; 13.77, 95% CI 13.24–14.29; and 2.58, 95% CI 2.43–2.73, respectively) and subway stations (16.89, 95% CI 7.96–25.82; 9.72, 95% CI 5.41–14.04; and 2.44, 95% CI 0.65–4.24, respectively). PFNSFs were scarce across all transportation facilities, with mean densities ranging from 0.06 to 0.14.

**Table 1:** Distribution of food retail establishments in the surroundings of bus stations, bus stops, and subway stations in the city of Belo Horizonte, Minas Gerais, according to the health vulnerability index (Brazil, 2019)

**Food retail establishments**	**Total**^**a**^ ***n* = 47**			**Health vulnerability index**^**b**^					
				**Low** ***n* = 34**		**Medium** ***n* = 0**		**High or very high** ***n* = 13**	
	**Mean**	**95% CI**	**Minimum–maximum**	**Mean**	**95% CI**	**Mean**	**95% CI**	**Mean**	**95% CI**
**Bus stations**									
Unprocessed and minimally processed foods	2.87	1.32–4.42	0–30	1.24	0.69–1.79	-	-	7.15	2.01–12.30
Mixed foods	14.94	9.69–20.19	0–76	7.41	5.30–9.53	-	-	34.62	20.51–48.72
Ultraprocessed foods	23.19	14.08–32.31	1–122	12.56	8.05–17.07	-	-	51.00	23.46–78.54
PFNSF	0.06	−0.01–0.14	0–1	0.09	−0.01–0.19	-	-	0	0–0
**Food retail establishments**	**Total**^**a**^ ***n* = 47.003**			**Health vulnerability index**^**b**^					
				**Low** ***n* = 1.323**		**Medium** ***n* = 1.432**		**High or very high** ***n* = 1.009**	
	**Mean**	**95% CI**	**Minimum–maximum**	**Mean**	**95% CI**	**Mean**	**95% CI**	**Mean**	**95% CI**
**Bus stops**									
Unprocessed and minimally processed	2.58	2.43–2.73	0–83	2.91	2.63–3.19	2.8	2.50–3.09	2.89	2.47–3.32
Mixed foods	13.77	13.24–14.29	0–174	14.63	13.62–15.64	14.45	13.43–15.47	16.12	14.71–17.53
Ultraprocessed foods	14.21	13.67–14.75	0–145	15.74	14.64–16.85	14.82	13.79–15.84	17.18	15.71–18.65
PFNSF	0.14	0.13–0.16	0–3	0.15	0.13–0.18	0.15	0.13–0.17	0.16	0.13–0.19
**Food retail establishments**	**Total**^**a**^ ***n* = 18**			**Health vulnerability index**^**b**^					
				**Low** ***n* = 16**		**Medium** ***n* = 0**		**High or very high** ***n* = 0**	
	**Mean**	**95% CI**	**Minimum–maximum**	**Mean**	**95% CI**	**Mean**	**95% CI**	**Mean**	**95% CI**
**Subway stations**									
Unprocessed and minimally processed	2.44	0.65–4.24	0–14	2.69	0.68–4.70	-	-	-	-
Mixed foods	9.72	5.41–14.04	0–27	10.69	6.03–15.34	-	-	-	-
Ultraprocessed foods	16.89	7.96–25.82	1–61	18.63	8.85–28.40	-	-	-	-
PFNSF	0.06	−0.06–0.17	0–1	0.06	−0.07–0.20	-	-	-	-

Note: Mean number of establishments located within a 500-m circular buffer surrounding each public transportation facility

*PFNSFs* public food and nutrition security facilities

^a^Considering all 5017 census tracts

^b^Considering the 3830 census tracts with available information on the health vulnerability index (IVS)

Higher levels of social vulnerability generally corresponded to higher mean densities of establishments predominantly selling ultraprocessed foods. Around bus stations, mean densities increased from 12.56 (95% CI 8.05–17.07) in low-vulnerability areas to 51.00 (95% CI 23.46–78.54) in high and very high vulnerability areas. Around bus stops, densities increased from 15.74 (95% CI 14.64–16.85) to 17.18 (95% CI 15.71–18.65), whereas the availability of establishments selling unprocessed or minimally processed foods remained relatively stable across vulnerability levels. PFNSFs were absent around bus stations in high-vulnerability areas and showed little variation across vulnerability levels around bus stops (Table 1).

## Discussion

This study showed that public transportation facilities in Belo Horizonte are predominantly surrounded by establishments selling ultraprocessed foods, whereas establishments selling unprocessed or minimally processed foods and PFNSFs were scarce. These findings are consistent with previous studies conducted in São Paulo and the Rio de Janeiro metropolitan region, which also reported the predominance of unhealthy food outlets in transportation settings [2, 6]. Together, these findings suggest that public transportation facilities represent obesogenic urban environments that may influence food purchasing during daily commuting.

A novel contribution of this study is the incorporation of social vulnerability into the assessment of food environments surrounding transportation facilities. Although establishments selling unprocessed or minimally processed foods were also identified in more vulnerable areas, these locations consistently presented greater availability of establishments selling ultraprocessed foods, particularly around bus stations. Because public transportation is frequently used by populations experiencing greater social vulnerability, this pattern may increase daily exposure to unhealthy food environments and reinforce existing inequalities in food access. Although the present study was descriptive, these findings suggest that future analytical studies should further investigate the relationship between transportation infrastructure, food environments, and social vulnerability.

These findings have important implications for urban health and food policy. Public transportation facilities are high-traffic urban spaces that may substantially influence daily food exposure, particularly among populations living in socially vulnerable areas who rely more heavily on public transit. The predominance of establishments selling ultraprocessed foods, together with the limited presence of PFNSF, suggests that these environments may reinforce existing inequalities in access to healthy foods and undermine the principles of food and nutrition security [3, 11]. This food environment may also contribute to unhealthy dietary patterns and, consequently, to the growing burden of diet-related noncommunicable diseases, particularly among populations facing socioeconomic and territorial barriers to accessing healthy foods [7, 12]. These findings also highlight an important policy gap, as public transportation facilities remain largely overlooked by food environment interventions despite their strategic role in urban mobility. Expanding PFNSF, farmers’ markets, and other healthy food initiatives in these locations, combined with regulatory measures to limit the availability and marketing of ultraprocessed foods, could strengthen healthier food environments and reduce inequalities in food access [3, 4, 11, 23, 24].

This study has some limitations. First, analyses were restricted to formal food retailers and did not include informal vendors, who are common around Brazilian transportation facilities and frequently sell ultraprocessed foods. Consequently, exposure to unhealthy food environments may have been underestimated. Second, the limited number of bus and subway stations reduced the precision of estimates for these facilities. Finally, the HVI was based on the 2010 Brazilian Census, whereas food environment data referred to 2019, which may have resulted in some misclassification of vulnerability levels. Nevertheless, the HVI remains the official and most comprehensive indicator available for assessing intraurban social vulnerability in Belo Horizonte.

In conclusion, public transportation facilities in Belo Horizonte are characterized by a predominance of establishments selling ultraprocessed foods and a limited presence of healthy food retailers and PFNSF, particularly in socially vulnerable areas. Recognizing these locations as strategic settings for urban food policies may help reduce inequalities in food access and promote healthier urban environments.

## Data Availability

The data used in this study were obtained from publicly available sources. Data on food retail establishments are available from the Secretariat of Finance of the State of Minas Gerais (Secretaria de Estado de Fazenda de Minas Gerais) upon request. Data on public food and nutrition security facilities, bus stops, bus stations, and subway stations are publicly available through the Belo Horizonte Municipal Government website. The datasets generated and analyzed during the current study are available from the corresponding author upon reasonable request.
